# Practical Notes on Diagnosis and Treatment

**Published:** 1907-10-05

**Authors:** 


### Practical Notes on Diagnosis and Treatment.

**Ice in Lumbago.**

Dr. Kinnear claims good results in lumbago from the application of an ice bag. He says that the treatment will readily cut short an acute attack, and will immediately comfort the patient.

**Hæmorrhage from the Umbilical Cord.**

In a case of hæmorrhage from the umbilical cord Dr. Geo. W. R. Dabbs painted the stump with adrenalin 5 parts and vernisol 95 parts, with completely satisfactory results. Three earlier children had all died from hæmorrhage on separation of the cord.

**Sarcoma of the Kidney.**

In sarcoma of the kidney in early life hæmaturia seldom occurs, and, in fact, there is, as a rule, no alteration in the urine which would point to the serious nature of the disease in the kidney. The enlargement is painless, and beyond the steady growth of the tumour, and the gradual loss of flesh and strength of the patient, there are few symptoms to chronicle.—*Mr. Mayo Robson.*

**Phimosis.**

When a chronic foul-smelling discharge is found exuding from beneath a phimosed foreskin it should lead to a suspicion that the malady may be malignant, and the foreskin should be slit up and the parts carefully examined.—*Sir Wm. MacCormac.*

**Hæmorrhage or Thrombosis.**

The initial depression of the bodily temperature is far more marked in encephalic hæmorrhage than in either thrombosis or embolism. Practically, apart from hæmorrhage, a lowering of the temperature below 96° is only observed in thrombosis of the basilar artery.—*Dr. Charlton Bastian.*

**Laryngeal Paralysis and Lead.**

Some cases of paralysis of one vocal cord have been described as due to chronic lead poisoning. Naunyn states that in horses which work in lead mills paralysis of the abductors of the vocal cords is common, and that not infrequently this necessitates tracheotomy.—*Dr. Vivian Poore.*

